# Development of Cynicism in Medical Students: Exploring the Role of Signature Character Strengths and Well-Being

**DOI:** 10.3389/fpsyg.2020.00328

**Published:** 2020-02-27

**Authors:** Timo Kachel, Alexandra Huber, Cornelia Strecker, Thomas Höge, Stefan Höfer

**Affiliations:** ^1^Institute of Psychology, University of Innsbruck, Innsbruck, Austria; ^2^Department of Medical Psychology, Medical University Innsbruck, Innsbruck, Austria

**Keywords:** signature character strengths, burnout, cynicism, medical students, well-being, latent growth modeling (LGM), latent class growth analysis (LCGA), growth mixture modeling (GMM)

## Abstract

Reports of medical students experiencing burnout-related symptoms (e.g., cynicism) have increased in recent years. Little is known about the developmental process of this phenomenon and its relations with signature character strengths and well-being. The aim of this longitudinal analysis was to explore changes in the level of cynicism of medical students while in preclinical education. We further examined how the applicability of signature character strengths and well-being are related to this developmental process. Medical students (*N* = 99) participated in three online surveys over 3 years during medical school. Latent growth modeling, latent class growth modeling, general mixed modeling was conducted, and *post hoc* mixed ANOVA, Friedman test and Welch test analyses were examined. The results showed an increase in cynicism among medical students from first to last measurement. Two groups with distinct developmental trajectory patterns of cynicism were identified. Students with high levels of cynicism (*high-level* group) and students with changing levels of cynicism (*increasing* group) perceived higher applicability of signature character strengths in private life compared to the study context. Moreover, the *high-level* group experienced significantly lower psychological well-being (in particular mastery, optimism, and relationship) in their first year of medical education. This explorative study offers a comprehensive understanding of cynicism development in medical students during medical school and its relations to the applicability of signature character strengths and well-being. Prospective replication studies are needed to replicate the results obtained in this study.

## Introduction

Medical universities are responsible for theoretical and practical scientific education, aiming to train future physicians to become professional, skillful and occupationally competent graduates. Therefore, universities provide a curriculum offering diverse methods such as lectures, supervised practice mentoring, and communication training in order to reach these goals. For a considerable number of medical students, this longstanding training process takes its toll regarding their physical health (e.g., infections due to exposure to bacteria or viruses in hospitals), mental health (e.g., Brazeau et al., 2014), and well-being (e.g., Dunn et al., 2008).

One of the continuous major concerns of researchers is the development of burnout during the time in medical school (e.g., Dahlin and Runeson, 2007; Dyrbye and Shanafelt, 2016), which is associated with several consequences affecting health, such as alcohol abuse (Jackson et al., 2016) and suicidal ideation (Dyrbye et al., 2008; Kosik et al., 2017). According to Dyrbye et al. (2014), 56% of medical students experienced symptoms of burnout and over 11% reported suicidal thoughts (Rotenstein et al., 2016). Furthermore, a meta-analysis by Frajerman et al. (2018) exposed that burnout is prevalent among 44.2% of medical students. In line with these results, Prinz et al. (2012) described similar findings for a German sample. Burnout among students is considered a three-dimensional psychological syndrome of emotional exhaustion, cynicism, and reduced personal accomplishment, which results in the deterioration of academic engagement (Schaufeli et al., 2002b). Furthermore, it plays a potentially important role in predicting future burnout after becoming a graduated professional (Robins et al., 2017). Emotional exhaustion and cynicism are often perceived as the main dimensions of burnout (Mäkikangas and Kinnunen, 2016), whereas reduced personal accomplishment was not as strongly related to these two burnout components, and therefore challenged for its questionable validity (Bresó et al., 2007; Schaufeli and Salanova, 2007).

Maslach and Leiter (2016) criticized the approach of solely reducing burnout to the dimension of emotional exhaustion, as it tends to be done increasingly nowadays. According to Leiter and Maslach (2016), cynicism does play a crucial role in work environments, as it reflects “the poor quality of social relationships at work and the lack of critical resources” (p. 98). Furthermore, the substance of cynicism has been emphasized, as it seems to have a more important role in the burnout experience than emotional exhaustion alone, which is why the authors suggested that further analyses should pay more attention to the dimension of cynicism (Leiter and Maslach, 2016). “The cynicism dimension was originally called depersonalization (given the nature of human services occupations), but was also described as negative or inappropriate attitudes toward clients, irritability, loss of idealism, and withdrawal” (Maslach and Leiter, 2016, p. 103). Regarding the medical education process, cynicism is more of a distant or indifferent attitude toward one’s own studies and their potential usefulness.

Many researchers have described the increase of cynicism beginning in medical school (e.g., Wolf et al., 1989; Dyrbye et al., 2005) and progressing during residency (Collier et al., 2002). One might suppose that most medical students start their training with the idea of helping people (Pagnin et al., 2013) and doing the “right thing,” which is reflected in some sense of idealism. Although this attitude seems to be noble, it will not last for a long time (Morley et al., 2013). Fading idealism is associated with increased weariness concerning the medical profession, reduced interest in helping underserved people and reduced feelings of responsibility for the health of society in general (Crandall et al., 1993; Woloschuk et al., 2004; Smith and Weaver, 2006). The combined decline of idealism and increase of cynicism was initially described in the mid-1950s (Eron, 1955). Although decreases in empathy and growth of cynicism during medical school have been well narrated, there is still some debate on whether both processes occur as a result of training progress and its formal, informal and hidden curriculum (e.g., Feudtner et al., 1994; Hojat et al., 2009; Neumann et al., 2011). In general, cynicism was found to be related to declines in physical health, indicated by higher BMI scores and C-reactive protein levels, a decrease in heart rate variability and self-rated physical health (Viljoen and Claassen, 2017). Apart from the links with physical health, there are also drawbacks concerning the interpersonal and mental level. For example, a cynical attitude in the environment of medical training contributes to the erosion of professionalism (Dyrbye et al., 2005). Moreover, cynicism is crucial to the burnout experience, as it was found to predict turnover in the health care sector (Leiter and Maslach, 2009), and study-related cynicism forecasted work-related cynicism as noticed by a longitudinal analysis (Robins et al., 2017). Furthermore, a qualitative study by Peng et al. (2018) revealed that cynicism was associated with negative effects on physicians’ career satisfaction, quality of patient care and quality of mentorship for following generations. The development of cynicism in medical school is possibly associated with a depersonalized attitude as physician, which results in poorer communication skills (Brown et al., 2009), lower patient satisfaction or longer post-discharge recovery time (Halbesleben and Rathert, 2008). In contrast to these findings, cynicism may be seen to act as coping mechanism to reduce stress (McManus et al., 2002), handle new situations (Wolf et al., 1989) or create distance between oneself and the patients’ hardship (Kopelman, 1983; Peng et al., 2018).

The Job Demands-Resources Model by Bakker and Demerouti (2007) assumes that burnout occurs when people encounter an imbalance between job demands and resources. Therefore, high demands (e.g., work overload or time pressure) in work environments are risk factors for the development of health-related problems (e.g., symptoms of burnout). This can be reduced by job resources (e.g., supportive colleagues, autonomy) capable of dealing with such demands (Schaufeli and Bakker, 2004). This model has also shown to be applicable in the context of health professional studies (Gusy et al., 2016). Some researchers blame the unfavorable circumstances in the learning environment of medical education for the development of burnout (e.g., Dyrbye and Shanafelt, 2016). This environment is characterized by highly demanding study conditions, while condition-related resources often appear to lack (Dahlin et al., 2005). Although it might seem clear that structures and conditions should be changed in order to create an environment contributing to health, this process is not an easy one. Therefore, it is necessary to consider personal resources.

A study by Heinen et al. (2017), conducted with a sample of medical students, found that personal resources (e.g., optimism, self-efficacy, and resilient coping) can act as a buffer in the perception of study-related stressors and emotional distress. “Personal resources are aspects of the self that are generally linked to resiliency” (p. 632) and these resources are related to the individuals’ sense of their ability to master or influence their environment successfully (Hobfoll et al., 2003). According to Bandura (2000), environmental factors play a crucial role in nurturing the individual level of personal resources, and in turn, these resources are connected to people’s capability to perceive and adapt to their environment.

Character strengths as a concept of (Seligman and Csikszentmihalyi, 2000) positive psychology can be seen as such personal resources. They are components of the good character, morally valued, trait-like and can be facilitated by rituals or institutions (Peterson and Seligman, 2004). Furthermore, they are considered essential, as they relate to stress coping behavior in health care professionals (Harzer and Ruch, 2015). Character strengths have positive associations with overall perceived health (Proyer et al., 2014) and a unique value in predicting physical health (Hanks et al., 2014). Furthermore, physical and mental health were positively related to character strengths (Leontopoulou and Triliva, 2012). A study by Hausler et al. (2017b) found character strengths to be linked with well-being and mental health but not with physical health in a sample of medical students and resident physicians. Moreover, character strengths are related to physicians’ engagement and patient care (Seoane et al., 2016). Peterson and Seligman (2004) proposed that most people have 3–5 core or “signature” character strengths, which are typical of the respective individual. These signature character strengths are the ones a person “celebrates, and frequently exercises” and it is believed that their “exercise […] is fulfilling” (Peterson and Seligman, 2004, p. 18). Signature character strengths fulfill several criteria (Peterson and Seligman, 2004), e.g., “feeling of invigoration when using a signature strength” or “desire to act in accordance with the signature strength.” It might be assumed that the use of signature character strengths leads to better relations, positive emotions and meaning (Seligman, 2011). Several studies have shown that they can also contribute to physical and mental health, well-being, academic work and life satisfaction (Peterson and Seligman, 2004; Seligman et al., 2005; Peterson and Park, 2011; Allan and Duffy, 2014).

Besides the sole possession of character strengths, it is important to account for their applicability. According to Harzer and Ruch (2012, 2013) two conditions must be met to apply character strengths: Firstly, one’s possession of the character strength is crucial to show strength-related behavior. Secondly, circumstantial conditions (e.g., private life or workplace/studies) allow or call for the display of character strengths. Hence, the “Applicability of Character Strengths Rating Scale” ACS-RS (Harzer and Ruch, 2013) was developed to measure the individually perceived influence of four conditions: two external ones: (I) normative demands and (II) appropriateness of strength-related behavior in given situations; two internal ones: (III) perceived presence of factors that may facilitate or hamper strength-related behavior and (IV) intrinsic motivation to show certain behavior (Harzer and Ruch, 2012, 2013). Recently conducted cross-sectional studies with samples of medical students and physicians emphasized the important role of one’s applicable signature character strengths (applicability of signature character strengths in the context of study and work = ASCS-S/-W; and private life = ASCS-P) to foster mental health, well-being, burnout and work-engagement (Hausler et al., 2017a; Huber et al., 2019; Strecker et al., 2019). Furthermore, Höge et al. (2019) could demonstrate with a sample of hospital physicians that the ASCS-W at the first measurement (T1) had an impact on the perception of the socio-moral climate of the medical department at the second measurement (T2) and reversed effects of the socio-moral climate at T1 on the ASCS at work at T2. Moreover, cross-sectional results showed an indirect effect of the socio-moral climate on work engagement and psychological well-being via the ASCS at work.

Altogether, studies in the field of positive psychology have rarely focused on medical students. In particular, due to a lack of longitudinal data in this area, it merits investigating potential variables and their interrelations contributing to the health of medical students. Thus, this study takes a closer look at the following research gaps:

Firstly, there is no information regarding the homogeneity of the developmental process of cynicism in Austrian medical students. The authors assume that an increase in cynicism is prevalent among preclinical medical students during their time in medical school, which has also been shown in other studies with different samples.

Secondly, to the best of the authors’ knowledge there have been no studies so far exploring the developmental process of cynicism and possible differences among individuals. Therefore, this study investigated whether there are different trajectory patterns and how groups of students may differ from each other.

Thirdly, the applicability of signature character strengths could be a personal resource when it comes to predicting burnout-symptoms (e.g., Leiter et al., 2012). Although studies highlighted the relevance of the congruence between persons characteristics and university-related environmental fit on health outcomes (e.g., Jiang and Jiang, 2015), there is a research gap regarding the potential role of the applicability of signature character strengths on the development of cynicism during medical school. Thus, associations between the applicability of signature character strengths and developmental patterns of cynicism were analyzed. Furthermore, we examined the relationship between subjective well-being, psychological well-being (autonomy, engagement, mastery, meaning, optimism, and relationship) and trajectory patterns of cynicism.

## Materials and Methods

### Participants and Procedure

The longitudinal data collection was conducted between January 2015 and March 2017 at a medical university in Austria. The institutional review board gave approval and participants completed online questionnaires after being invited via email at three time points. Offered incentives were medical education credits, as well as a raffle of medical books relevant to their current semester. Of all 504 participating medical students, 85.5% participated at the first (*N1* = 431), 53% at the second (*N2* = 267), and *N3* = 101 (20%) at all three measurements. We only used complete data sets and no data imputation methods were applied. After matching those students who participated in all three annual measurement waves and excluding two outliers with overall extreme response patterns regarding relevant variables (critical *z*-values ± 3.29; Osborne and Overbay, 2004; Tabachnick and Fidell, 2007), the final sample consisted of 99 medical students. At baseline measurement, the medical students’ mean age was 20.3 (*SD* = 2.1; range = 18–28) being in their first year of medical education. Nearly two thirds of the participants were female (63.6%), and 52.5% of the participating students were Austrians followed by 25% Germans. At baseline, 74.7% of medical students were not in a relationship and of all participants, 45.5% lived in a shared apartment. 34.5% of students invested at least 40 h per week into their studies. Having a part-time job, on which they spent between 2 and 16 h per week, was reported by 16%.

### Measures

The cynicism of medical students was measured with the cynicism-subscale of the German version of the “Maslach-Burnout-Inventory Student Survey” (MBI-SS-GV; Gumz et al., 2013; original: Schaufeli et al., 2002a). The cynicism-subscale consists of 4 items rated on a seven-point scale from 0 = *never* to 6 = *daily*, with higher values indicating more cynicism symptoms. An item example is: “I have become more cynical about the potential usefulness of my studies.” Cronbach’s alpha for the cynicism scale in this study was at T1 α = 0.76, at T2 α = 0.79, and at T3 α = 0.85.

Character strengths were measured with the “Values in Action Inventory of Strengths” (VIA-IS; Peterson et al., 2005). The original version of the VIA-IS has 240 Items, but for economic reasons the shorter validated 120-item German questionnaire (Höfer et al., 2019) was used to measure the 24 individual character strengths consisting of five items each, rated on a five-point scale from 1 = *strongly disagree* to 5 = *strongly agree*. Item examples are: “I am never too busy to help a friend” (kindness) or “I always keep my promises” (honesty). The German short-form has comparable psychometric properties to the 240-item version with Cronbach’s alpha ranging between α = 0.63 (honesty) and α = 0.90 (spirituality) (Höfer et al., 2019). For our sample the internal consistency was between α = 0.65 (teamwork) and α = 0.89 (spirituality). The five highest character strengths of each individual (so-called signature character strengths; Peterson and Seligman, 2004) were identified. Furthermore, following recommendations by Harzer and Ruch (2012), we considered candidates’ possessing signature character strengths, when the VIA-IS score was 3.5 or higher.

The “Applicability of Character Strengths Rating Scale” (ACS-RS; Harzer and Ruch, 2013) was used to assess the applicability of signature character strengths regarding private life (ASCS-P) and studies (ASCS-S) related conditions, independent of the possession of character strengths. The items ask if the character strength is “demanded,” “helpful,” “used,” and “important for me” in private life and studies. In the present dataset, the applicability of the five highest signature character strengths was measured after identifying them with the VIA-120. To assess the applicability, eight items (four referring to private life and the other four referring to studies) for each signature character strength were rated on a five-point scale ranging from 1 = *never* to 5 = *(almost) always*. Internal consistencies (median) were for ASCS-P at T1 α = 0.81, T2 α = 0.84, and T3 α = 0.87, for ASCS-S at T1 α = 0.76, T2 α = 0.77, and T3 α = 0.78.

Well-being was examined by using the “Comprehensive Inventory of Thriving” (CIT; Su et al., 2014). It measures seven components of well-being with 18 subscales three assigned to subjective well-being (SWB): life satisfaction, positive and negative feelings; 15 to psychological well-being (PWB): autonomy, engagement, mastery, meaning, optimism and relationship, composed of 54 items rated on a five-point scale ranging from 1 = *strongly disagree* to 5 = *strongly agree*. In addition to the calculation of a composite score of SWB and PWB, a general well-being (thriving) mean score can be computed. Item examples are: “I feel good most of the time” (SWB; positive feelings) or “I expect more good things in my life than bad” (PWB; optimism). The German version has been validated by Hausler et al. (2017), who demonstrated its reliability and validity. Internal consistencies for our sample ranged between α = 0.93 (T1), α = 0.92 (T2), and α = 0.95 (T3).

### Data Analysis

#### Development of Cynicism

In order to examine medical students’ trajectories of cynicism, latent growth models were applied (LGM) in Mplus Version 8.2 (Muthén and Muthén, 1998–2018). LGM is a modeling technique that is capable of capturing inter-individual (between-person) variability in intra-individual (within-person) patterns of change over time (Curran et al., 2010) via estimating growth parameters. For example, *intercept* is the average mean beginning value, and *slope* is the average rate of change (e.g., linear, quadratic). According to Hamilton et al. (2003) our sample size for this analysis is adequate. Due to robustness against non-independence and non-normality of the variables, the robust maximum likelihood estimation (MLR) was selected. The following fit indices were decisive for picking the best-fitting model: (a) non-significant chi-square statistic, the (b) root mean square error of approximation (RMSEA) with values ≤ 0.08, and the (c) comparative fit index (CFI) with values > 0.90, indicating satisfying model fit (Hoyle, 2012). The LGM with a linear slope term (i.e., assuming there is a linear growth in cynicism over time) was compared to the intercept-only model (i.e., assuming there is a stable overall level of cynicism over time) by using the Satorra–Bentler scaled chi-square difference test (Satorra and Bentler, 2001).

#### Capturing Heterogeneity in the Development of Cynicism

Since a conventional LGM is only capable of estimating one set of growth parameters for an entire sample, it is not possible to capture “subgroups” of people who may deviate from that average-level trajectory. Therefore, growth mixture modeling (GMM; Muthén, 2004) and latent class growth analyses (LCGA; Nagin, 2005), as exploratory data driven processes, were conducted. GMM assumes that populations can be divided into subpopulations or “classes,” each following their own distribution (Muthén, 2004). Therefore, each class has its own set of growth parameters which may be allowed to vary within and between classes. Loosely speaking, a one-class GMM with two growth parameters (i.e., intercept, linear) is in essence a conventional LGM. LCGA – as a special type of GMM – on the other hand allows no within class variability of the growth parameters, which means it is assumed that individuals within these classes are taxonomically similar regarding their outcome variables (Nagin and Odgers, 2010; Frankfurt et al., 2016).

Both methods (LCGA and GMM) assume that the number of trajectory patterns is unknown but finite. Due to this assumption, it is necessary to choose the number of classes beforehand and use a combination of several criteria to validate the given solution. LCGA can be used as first exploratory step, prior to a GMM analysis, to identify the number of classes for further analyses (Jung and Wickrama, 2008). Furthermore, LCGA is usually more “robust” to convergence problems often appearing in GMM, since it estimates fewer parameters. To face estimation problems in LCGA and GMM (e.g., finding local maxima), it is recommended to set the number of random sets of starting values for analyzed mixture models between 400 and 1000 (MPlus version 8.2; default = 20) and the number of optimizations in the final stage between 100 and 250 (MPlus version 8.2; default = 4). For the bootstrapped generated data, the number of random sets of starting values and the optimizations in the final stage for the *k*-class model are set to 40 respectively 8 by default in MPlus version 8.2. However, setting the value of these numbers for the analyzed and bootstrapped model mainly depends on whether replicated solutions are obtained successfully. For our analyses we chose 1000 random sets of starting values and 250 optimizations in the final stage for the analyzed model and 500 random sets of starting values and 200 optimizations in the final stage for the generated bootstrapped *k*-class model. Moreover, to ensure sufficient precision of the estimated *p*-value of the bootstrapped likelihood ratio test (BLRT) we selected 250 bootstrap-samples.

Although there is no standard procedure in identifying the “most suitable” model with an explicit number of classes, we applied eight criteria to determine the best solution: (a) the Bayesian information criterion (BIC; Schwarz, 1978), as commonly used model fit indicator, should be smaller if model fit improves; (b) sample-size-adjusted BIC (aBIC) is a more precise fit index for small sample sizes (Henson et al., 2007); (c) the Vuong–Lo–Mendell–Rubin likelihood ratio test (VLMR-LRT; Vuong, 1989; Lo et al., 2001) compares the *k*-1 versus the *k*-class solution (e.g., 2- vs. 3-classes) and indicates via significance an improved fit of the *k*-class solution; (d) the adjusted Lo–Mendell–Rubin likelihood ratio test (adj. LMR-LRT; Muthén, 2004) tends to produce fewer type I errors (Henson et al., 2007); (e) the bootstrapped likelihood ratio test (BLRT; McLachlan and Peel, 2000) is used to double-check the LRT solution, since it has proven to be significantly better (Nylund et al., 2007); (f) the entropy value ranges from 0 to 1 and is available for models with at least two classes. Entropy does not serve as a fit index as it is a measure for individual classification accuracy. Values of 0.75 or higher are acceptable (Reinecke, 2006); (g) each class should at least comprise 5% of the sample (Nelemans et al., 2014), although we tightened this prerequisite, as each group should include at least ten students (10.1%); (h) and finally deciding on the model with parsimonious properties (i.e., fewer number of parameters).

The chosen best-fitting model was further examined with mixed ANOVA testing and *post hoc* analyses regarding class differences in cynicism, applicability of signature character strengths and well-being components. For violated sphericity assumptions Huynh–Feldt correction was used, if eta (ε) was > 0.75. *Post hoc* analyses were corrected by Bonferroni-method in order to account for alpha error inflation. For this purpose, IBM SPSS Statistics 24 (IBM Corporation, 2016) was used.

## Results

### Descriptive Statistics

Table 1 presents the (inter-) correlations, means, standard deviations and internal consistencies of all variables over the period of the three annual measurement waves. Pearson correlations of cynicism (*r* = 0.31 to *r* = 0.65) and SWB (*r* = 0.38 to *r* = 0.63) vary strongly between successive waves, indicating large individual differences in the stability of cynicism and SWB over time. It can also be noted that the mean value of cynicism increases over time. There was a higher stability for ASCS-S (*r* = 0.42 to *r* = 0.60), ASCS-P (*r* = 0.46 to *r* = 0.61) and PWB (*r* = 0.66 to *r* = 0.77). Significant correlations can be found between cynicism and the applicability of signature character strengths in private life at the first (*r* = −0.29), second (*r* = −0.21) and between the first and second measurement (*r* = −0.32). Furthermore, ASCS-S is significantly associated with cynicism over the whole measurement period, ranging between *r* = −0.23 and *r* = −0.38. Negative correlations are shown between cynicism and SWB (*r* = −0.21 to *r* = −0.35), and even more strongly between cynicism and PWB (*r* = −0.26 to *r* = −0.49).

**TABLE 1:** (Inter-) correlations, means, standard deviations and internal consistencies among the variables of the study population (*N* = 99).

Measure	1	2	3	4	5	6	7	8	9	10	11	12	13	14	15	16
1. ASCS-P, T1	(–)															
2. ASCS-P, T2	0.48**	(–)														
3. ASCS-P, T3	0.61**	0.46**	(–)													
4. ASCS-S, T1	0.39**	0.28**	0.35**	(–)												
5. ASCS-S, T2	0.21*	0.56**	0.25*	0.45**	(–)											
6. ASCS-S, T3	0.21*	0.27**	0.44**	0.60**	0.42**	(–)										
7. Cynicism, T1	−0.29**	−0.32**	–0.11	−0.25*	−0.23*	–0.15	(–)									
8. Cynicism, T2	–0.05	−0.21*	0.05	–0.18	−0.38**	−0.26**	0.34**	(–)								
9. Cynicism, T3	0.00	–0.14	0.09	–0.15	−0.29**	−0.36**	0.31**	0.65**	(–)							
10. SWB, T1	0.34**	0.32**	0.27**	0.22*	0.16	0.18	−0.35**	−0.27*	–0.13	(–)						
11. SWB, T2	0.15	0.42**	0.19	0.07	0.32**	0.28**	−0.21*	−0.37**	−0.25*	0.51**	(–)					
12. SWB, T3	0.09	0.20*	0.14	0.14	0.18	0.35**	–0.17	−0.25*	−0.23**	0.40**	0.63**	(–)				
13. PWB, T1	0.48**	0.48**	0.41**	0.45**	0.39**	0.40**	−0.49**	−0.29**	−0.26*	0.63**	0.45**	0.38**	(–)			
14. PWB, T2	0.26**	0.54**	0.26**	0.28**	0.51**	0.39**	−0.30**	−0.47**	−0.39**	0.48**	0.73**	0.52**	0.71**	(–)		
15. PWB, T3	0.29**	0.38**	0.32**	0.37**	0.38**	0.53**	−0.30**	−0.38**	−0.42**	0.46**	0.59**	0.69**	0.66**	0.77**	(–)	
16. Gender	0.00	0.06	0.08	0.05	0.07	0.06	0.03	0.01	0.04	0.08	0.03	0.07	0.06	0.04	0.03	(–)
α	0.78 –0.89	0.85 –0.91	0.81 –0.89	0.79 –0.92	0.76 –0.86	0.78 –0.87	0.73	0.79	0.85	0.94	0.93	0.92	0.91	0.93	0.94	(–)
*M*	4.24	4.30	4.29	3.79	3.81	3.75	0.97	1.25	1.63	4.06	4.00	4.03	4.01	3.98	3.95	0.36
*SD*	0.46	0.44	0.45	0.50	0.49	0.47	0.98	1.00	1.36	0.65	0.65	0.62	0.38	0.42	0.45	0.48

ASCS-P/-S, applicability of signature character strengths in private life/during studies; α = Cronbachs alpha; *M* = mean; *SD* = standard deviation; *p < 0.05, **p < 0.01; Gender (0 = female, 1 = male).

### Growth Analysis of Cynicism

The next step of our analysis was to determine if the model that assumes no linear growth (intercept-only model) fits better than the model supposing linear growth (linear model) in cynicism over three measurement points. Comparing the intercept-only model with the linear LGM of cynicism [Δχ^2^_SB_ (3) = 60.67, *p* < 0.01] revealed a better fit for the linear model (χ^2^ (1) = 0.54, *p* = 0.46, CFI = 1.00, RMSEA ≤ 0.001, 90% CI = [0.00, 0.24]). Medical students reported an initial intercept mean of 0.95 (*p* < 0.01) and an increasing linear slope mean of 0.33 (*p* < 0.01). Variances of intercept and slope were both significant (*p* < 0.01), indicating individual differences in starting values and rate of change in cynicism.

### Latent Class Growth Analysis and Growth Mixture Modeling Comparison (LCGA vs. GMM)

All tested models and their properties are depicted in Tables 2, 3. As an initial exploratory step, LCGAs were applied to the data and up to five classes were tested until converge issues occurred. Since likelihood ratio test statistics and entropy values are not available for one-class solutions, the empty cells were filled with dashes. From all tested LCGA-models, the one with the three-class solution provided the best fit according to BIC, aBIC, LMR and entropy. Next, we conducted GMM analyses with the variances of the intercept to be estimated (GMM^I^). Although the BIC and aBIC suggested a slightly better fit of the three-class model, taking also the LRTs into account, a better fit of the two-class solution was evident. We then further examined GMM solutions that allowed freely varying linear slope growth parameters (GMM^L^). Here, the best fitting model had two classes. Lastly, we compared a final set of different GMMs, with estimated intercept and linear slope growth parameters (GMM^IL^). According to the fit statistics, the two-class model provided the best fit. After an overall comparison between all tested models (LCGA, GMM^I^, GMM^L^ and GMM^IL^) regarding the eight mentioned criteria above (a–h), and due to the significant variances of intercept and slope of the initially tested linear LGM, the GMM^IL^ with two classes was chosen. It was most appropriate for identifying group differences in trajectories of cynicism.

**TABLE 2:** Parameters of model fit of all tested models.

Statistics	1 Class	2 Classes	3 Classes	4 Classes	5 Classes
**LCGA**					
BIC	888.63	825.88	811.42	816.66	814.24
aBIC	872.84	800.62	776.68	772.44	760.55
VLMR-LRT (*p*)	(–)	<0.01**	<0.01**	0.26	0.63
Adj. LMR-LRT (*p*)	(–)	<0.01**	<0.01**	0.29	0.65
BLRT (*p*)	(–)	<0.01**	1	1	1
Entropy	(–)	0.81	0.89	0.81	0.88
**GMM^*I*^**					
BIC	835.23	815.51	809.91	814.81	809.92
aBIC	816.28	787.09	772.01	767.44	753.08
VLMR-LRT (*p*)	(–)	<0.01**	0.29	0.23	0.04*
Adj. LMR-LRT (*p*)	(–)	<0.01**	0.31	0.24	0.06
BLRT (*p*)	(–)	<0.01**	1	1	1
Entropy	(–)	0.77	0.90	0.87	0.91
**GMM^L^**					
BIC	831.13	808.57	811.62	814.32	818.80
aBIC	812.18	780.15	773.72	766.95	761.96
VLMR-LRT (*p*)	(–)	0.02*	0.39	0.22	0.75
Adj. LMR-LRT (*p*)	(–)	0.02*	0.41	0.24	0.76
BLRT (*p*)	(–)	<0.01**	1	1	1
Entropy	(–)	0.84	0.84	0.86	0.87
**GMM^IL^**					
BIC	819.74	**807.14**	808.11	816.07	817.38
aBIC	794.48	**772.40**	763.90	762.39	754.22
VLMR-LRT (*p*)	(–)	0.02*	0.03*	0.71	0.20
Adj. LMR-LRT (*p*)	(–)	0.03*	0.04*	0.72	0.21
BLRT (*p*)	(–)	<0.01**	1	1	1
Entropy	(–)	**0.93**	0.93	0.87	0.91

The chosen model for the trajectories of cynicism is bold. LCGA, latent class growth analysis; GMM^*I*^ = growth mixture model with estimated intercept variances; GMM^*L*^ = growth mixture model with estimated linear slope variances; GMM^*IL*^ = growth mixture model with estimated intercept and linear slope variances; BIC = Bayesian information criterion; aBIC = sample size adjusted Bayesian information criterion; VLMR-LRT = Vuong-Lo-Mendell-Rubin likelihood ratio test; adj. LMR-LRT = adjusted Lo-Mendell-Rubin likelihood ratio test; BLRT = bootstrapped likelihood ratio test. *p < 0.05, **p < 0.01.

**TABLE 3:** Models and corresponsive proportion of each class.

**Class**		**Proportion of each class (%)**				**Model**
		
	1	2	3	4	5	

1	100	(–)	(–)	(–)	(–)	
2	56.4	43.6	(–)	(–)	(–)	
3	41.4	51.1	7.5	(–)	(–)	LCGA
4	14.6	50.5	7.1	27.8	(–)	
5	5.2	15.1	17.5	22.7	39.5	

1	100	(–)	(–)	(–)	(–)	
2	18.9	81.1	(–)	(–)	(–)	
3	6.5	39.1	54.4	(–)	(–)	GMM^I^
4	18.3	46.7	30.4	4.6	(–)	
5	15.9	9.7	16.8	22.1	35.5	

1	100	(–)	(–)	(–)	(–)	
2	35.9	64.1	(–)	(–)	(–)	
3	11.9	59.3	28.8	(–)	(–)	GMM^L^
4	9.1	24.5	49.4	17.0	(–)	
5	23.0	4.7	18.1	15.0	39.2	

1	100	(–)	(–)	(–)	(–)	
**2**	**66.0**	**34.0**	**(–)**	**(–)**	**(–)**	
3	6.1	64.6	29.3	(–)	(–)	**GMM^IL^**
4	6.2	29.0	9.5	55.3	(–)	
5	27.9	6.2	9.9	52.5	3.5	

*The chosen model for the trajectories of cynicism is bold. LCGA = latent class growth analysis; GMM^*I*^ = growth mixture model with estimated intercept variances; GMM^*L*^ = growth mixture model with estimated slope variances; GMM^*IL*^ = growth mixture model with estimated intercept and slope variances.*

**FIGURE 1:**
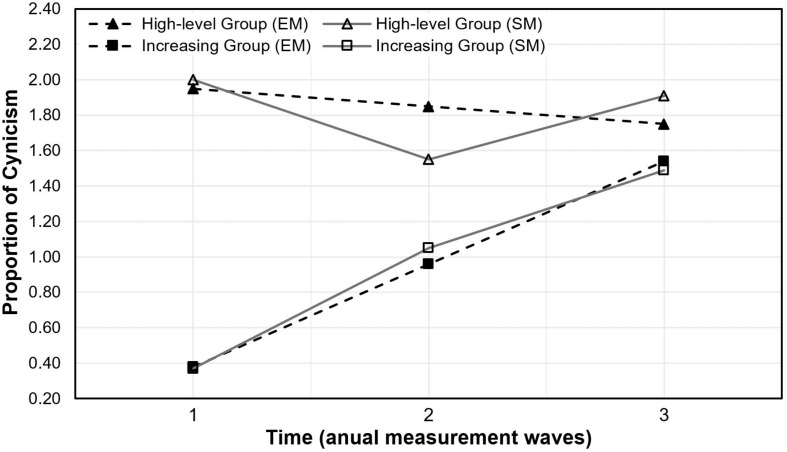
Two classes for trajectories of cynicism from the first to the third measurement. The high-level group is composed of 34% of the sample; the increasing group is composed of 66% of the sample. EM = estimated means; SM = sample means.

**FIGURE 2:**
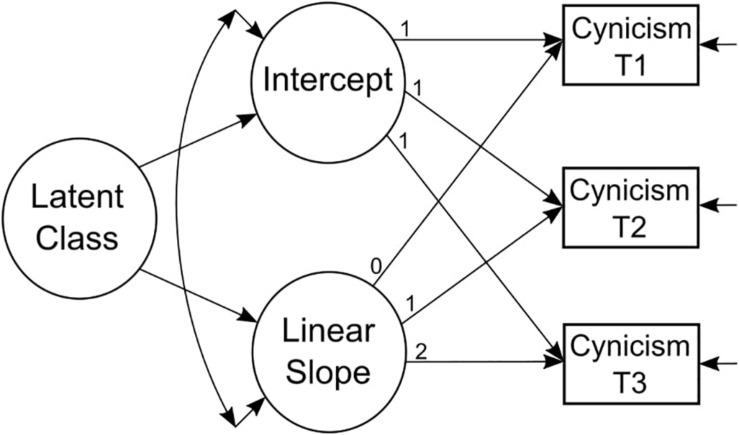
Growth mixture model (GMM) examines the development of cynicism in medical students from first to third year in medical training.

Figure 1 shows the classification solution of the GMM^IL^ with two trajectories of cynicism. Figure 2 shows a general depiction of a GMM. The first group, labeled as *high-level* group, consisted of 34% of the students and was described by an initially relatively high level of cynicism, with a significant 22.5% decrease after 1 year in medical training returning to the initial level at 3 years. The second group comprised 66% of the sample and was labeled *increasing* group, due to the relatively strong increase in cynicism over 3 years of medical training.

### *Post hoc* Analyses of the Classification Result

Table 4 shows the mixed ANOVA results with interaction and main effects. Table 5 shows means, standard deviations, repeated measures ANOVA and Friedman test results. In each class, 36.4% were male and 63.6% female. Both the *high-level* and the *increasing* group comprised 24.2% medical students in a relationship (T1). At initial measurement, 36.4% of the students in the *high-level* group and 50% in the increasing group lived in a shared apartment. Students of the *increasing* group were more diligent regarding their academic work, as 39.4% of them invested at least 40 h per week into their studies (T1) compared to the *high-level* group (24.2%). On the other hand, compared to the *increasing* group, the *high-level* group had an additional 5% of students, who had a side job. Furthermore, the *high-level* group experienced a higher workload in their part-time employment, since 45.4% had to work 10 h or more, in contrast to the *increasing* group (33.1%).

**TABLE 4:** Mixed ANOVA results with interaction and main effects.

Measure	Interaction			Main effect			Main effect		
	(Class × Time)			(Class)			(Time)		
			
	*F*	*p*	partial η^2^	*F*	*p*	partial η^2^	*F*	*p*	partial η^2^
**ACS-RS**									
ASCS-P	2.71	0.070	0.027	1.59	0.210	0.016	2.01	0.137	0.020
ASCS-S	0.56	0.575	0.006	1.00	0.320	0.010	0.52	0.594	0.005
**CIT**									
SWB	0.30	0.731	0.003	1.45	0.232	0.015	0.27	0.756	0.003
PWB	2.18	0.118	0.022	3.68	0.058	0.040	0.49	0.616	0.008
Autonomy	0.08	0.928	0.001	0.00	0.968	0.000	0.69	0.501	0.007
Engagement	0.18	0.398	0.009	0.84	0.360	0.009	1.90	0.152	0.019
Mastery	6.98	0.070	0.027	**4.55**	**0.035**	**0.045**	0.05	0.951	0.001
Meaning	0.03	0.975	0.000	1.71	0.195	0.017	0.09	0.915	0.001
Optimism	2.10	0.125	0.021	1.94	0.167	0.020	0.28	0.753	0.003
Relationship	1.13	0.330	0.012	3.15	0.079	0.031	2.04	0.132	0.021
**MBI-SS-GV**									
Cynicism	**18.98**	**<0.001**	**0.164**	**26.40**	**<0.001**	**0.214**	**12.07**	**<0.001**	**0.111**

*Significant mixed ANOVA interactions and main effects are bold. ASCS-P/-S = applicability of signature character strengths in private life/during studies; SWB = subjective well-being; PWB = psychological well-being.*

**TABLE 5:** Means, standard deviations (repeated measures) ANOVA and Friedman test results of the two classified cynicism groups.

Measure	Class 1 (High-level)			Class 2 (Increasing)		
		
	T1	T2	T3	T1	T2	T3

	*M | SD*	*M | SD*	*M | SD*	*M | SD*	*M | SD*	*M | SD*
**ACS-RS**						
ASCS-P	4.10^c^ | 0.53	4.22 | 0.56	4.30^c^ | 0.46	4.31 | 0.40	4.33 | 0.37	4.29 | 0.45
ASCS-S	3.70 | 0.42	3.76 | 0.51	3.73 | 0.49	3.84 | 0.54	3.83 | 0.48	3.76 | 0.46
**CIT**						
SWB	3.93 | 0.54	3.92 | 0.66	3.97 | 0.66	4.12 | 0.70	4.03 | 0.65	4.07 | 0.60
PWB	3.85^§^ | 0.37	3.89 | 0.44	3.88 | 0.50	4.09^§^ | 0.35	4.02 | 0.40	3.98 | 0.42
Autonomy	4.29 | 0.81	4.34 | 0.71	4.22 | 0.81	4.34 | 0.77	4.31 | 0.80	4.22 | 0.83
Engagement	3.74 | 0.75	3.93 | 0.76	3.92 | 0.86	3.95 | 0.66	4.01 | 0.61	3.97 | 0.61
Mastery	3.90^§^ | 0.42	3.98 | 0.52	3.99 | 0.54	4.21^§^ | 0.46	4.12 | 0.47	4.09 | 0.48
Meaning	3.81 | 0.88	3.79 | 0.95	3.76 | 0.99	4.01 | 0.96	4.01 | 0.88	3.99 | 0.80
Optimism	3.99^§^ | 0.69	4.02 | 0.76	4.11 | 0.69	4.30^§^ | 0.63	4.17 | 0.61	4.15 | 0.70
Relationship	3.73^§^ | 0.48	3.74 | 0.48	3.71 | 0.56	3.96^*c*§^ | 0.39	3.87 | 0.46	3.82^c^ | 0.49
**MBI-SS-GV**						
Cynicism	2.00^a§^ | 0.47	1.55^a†^ | 0.99	1.91 | 1.33	0.37^ac§^ | 0.38	1.05^ab†^ | 0.91	1.49^bc^ | 1.36

*Detected significant differences by repeated measures ANOVA or Friedman test are marked by alphabetic characters, ^*a*^ = T1 vs. T2, ^*b*^ = T2 vs. T3, ^*c*^ = T1 vs. T3; Significant differences by ANOVA (means between classes) are marked by symbols, ^§^ = Class 1 (T1) vs. Class 2 (T1), ^†^ = Class 1 (T2) vs. Class 2 (T2); ASCS-P/-S = applicability of signature character strengths in private life/during studies; SWB = subjective well-being; PWB = psychological well-being; M = mean; SD = standard deviation.*

We took a closer look at interaction and main effects between and within the *high-level* and the *increasing* group regarding cynicism. There was homogeneity of the error variances (Levene’s test; *p* > 0.05) and co-variances as assessed by Box’s test (*p* > 0.05). The Huynh–Feldt adjustment was used to correct for violations of sphericity (Mauchly’s test; *p* < 0.01). We found a statistically significant interaction between time and group [*F*(1.69, 164.11) = 18.98 *p* < 0.001, $\eta_{\text{p}}^{2}$ = 0.164), which can be seen in Figure 1. Differences between groups were significant at T1 [*F*(1, 97) = 339.92, *p* < 0.001, ω^2^ = 0.773] and T2 [*F*(1, 97) = 6.29, *p* = 0.014, ω^2^ = 0.051] with lower cynicism values in the *increasing* group. The cynicism score changes in the *increasing* group [Huynh–Feldt *F*(1.60, 103.78) = 42.24 *p* < 0.001, $\eta_{\text{p}}^{2}$ = 0.394] occurred at a significant level between all three measurements (Bonferroni corrected; *p* < 0.001) with increasing cynicism scores from the initial to the last measurement. The Bonferroni corrected *post hoc* analyses in the *high-level* group [*F*(2, 64) = 3.08, *p* = 0.049, $\eta_{\text{p}}^{2}$ = 0.089] were significant between T1 and T2 (*p* = 0.029) with decreasing scores in cynicism.

Regarding mixed ANOVA analyses for the applicability of signature character strengths outcome in private life (ASCS-P), we faced homogeneity of the error variances except for ASCS-P at T2 (Levene’s test; mean based *p* = 0.008). Conducting Box–Cox transformation did not yield a reasonable result (*p* = 0.035). Homogeneity of the co-variances (Box’s test; *p* = 0.004) was not present. Sphericity assumptions were not violated (Mauchly’s test; *p* > 0.05). Next, we analyzed differences within and between the two cynicism classes using a Friedman test and Welch tests. A Friedman test of differences among repeated measures was conducted and revealed a significant difference in ASCS-P depending on time point measured [χ^2^(2) = 8.99, *p* = 0.011] in the *high-level* group. *Post hoc* analysis was conducted and Bonferroni correction applied, resulting in significant differences between T1 and T3 (*p* = 0.011). The ASCS-P over the three annual measurements was stable in the *increasing* group. Welch tests did not report significant differences between both groups at any time point.

Concerning the testing for main effects and interaction regarding the applicability of signature character strengths during medical studies (ASCS-S), homogeneity of the error variances (Levene’s test; *p* > 0.05) and homogeneity of co-variances (Box’s test; *p* > 0.05) were assumed. Furthermore, there was no violation of sphericity (Mauchly’s test; *p* > 0.05). No statistically significant interaction between time and class [*F*(2, 194) = 0.56 *p* = 0.575, $\eta_{\text{p}}^{2}$ = 0.006] was perceived. Moreover, we found a main effect neither for time, nor for class. Although there was initially a slightly higher score in the *increasing* group, which decreased over the span of the three annual measurements, no statistically significant differences within and between both groups could be discovered. In general, the ASCS-P was distinctively higher than during studies in both groups and at all measurements.

Assumptions of sphericity for the mixed ANOVA of SWB had been violated (Mauchly’s test; *p* = 0.027). SWB showed homogeneity for the error variances (Levene’s test; *p* > 0.05) and homogeneity of co-variances (Box’s test; *p* > 0.05). Huynh–Feldt corrected results [*F*(1.92, 186.15) = 0.30 *p* = 0.731, $\eta_{\text{p}}^{2}$ = 0.003] did not show a significant interaction or main effect for time or class. Notwithstanding the fact that SWB in general tended to be lower in the *high-level* group compared with the *increasing* group, according to ANOVA test results, there was no significant difference within and between groups.

Mixed ANOVA testing with PWB demonstrated homogeneity of the error variances (Levene’s test; *p* > 0.05) and homogeneity of co-variances (Box’s test; *p* > 0.05). Sphericity assumptions had not been violated (Mauchly’s test; *p* > 0.05). Results neither revealed an interaction [*F*(2, 96) = 2.18 *p* = 0.118, $\eta_{\text{p}}^{2}$ = 0.022] nor main effects of class or time. Although PWB in general tended to be lower in the *high-level* compared with the *increasing* group, significant differences can only be found at T1 [*F*(1, 97) = 8.74, *p* = 0.004, ω^2^ = 0.073] with higher PWB values in the *increasing* group. Score changes of cynicism in the *increasing* group appeared to be significant [Huynh–Feldt *F*(1.83, 118.88) = 3.68 *p* = 0.032, $\eta_{\text{p}}^{2}$ = 0.054], but Bonferroni corrected *post hoc* analyses revealed no significant differences between time points (*p* > 0.05).

We further took a closer look at PWB components and conducted mixed ANOVA analyses. Autonomy, engagement, mastery, meaning, optimism and relationship showed homogeneity of the error variances (Levene’s test; *p* > 0.05) and assumptions of sphericity had not been violated (Mauchly’s test; *p* > 0.05). Heterogeneity of co-variances was present in engagement (Box’s test; *p* = 0.002), meaning (Box’s test; *p* = 0.023) and optimism (Box’s test; *p* = 0.034). Analyses did not show significant interactions for autonomy [*F*(2, 194) = 0.075 *p* = 0.93, $\eta_{\text{p}}^{2}$ = 0.001], engagement [*F*(2, 194) = 0.18 *p* = 0.398, $\eta_{\text{p}}^{2}$ = 0.009], mastery [*F*(2, 194) = 6.98, *p* = 0.07, $\eta_{\text{p}}^{2}$ = 0.027], meaning [*F*(2, 194) = 0.03, *p* = 0.975, $\eta_{\text{p}}^{2}$ < 0.001], optimism [*F*(2, 194) = 2.10 *p* = 0.125, $\eta_{\text{p}}^{2}$ = 0.02], and relationship [*F*(2, 194) = 1.13, *p* = 0.33, $\eta_{\text{p}}^{2}$ = 0.012]. For all components there were no main effects, except for mastery with a main effect for group [*F*(1, 97) = 4.55 *p* = 0.035, $\eta_{\text{p}}^{2}$ = 0.045]. Differences between *high-level* and *increasing* group were significant at T1 [*F*(1, 97) = 10.54, *p* = 0.002, ω^2^ = 0.088] with higher scores of mastery in the *increasing* group. Moreover, higher scores at T1 in the *increasing* group compared with the *high-level* group were also found for optimism [*F*(1, 97) = 5.06, *p* = 0.027, ω^2^ = 0.039], and relationship [*F*(1, 97) = 6.20, *p* = 0.014, ω^2^ = 0.05]. Autonomy, engagement, mastery, meaning and optimism were not significantly different between measurement waves within *high-level* and *increasing* group. Score differences between measurements of relationship appeared in the *increasing* group [*F*(2, 130) = 4.68, *p* = 0.011, $\eta_{\text{p}}^{2}$ = 0.067], *post hoc* analysis proved decreasing values between T1 and T3 (Bonferroni corrected; *p* = 0.02).

## Discussion

This study was set out to assess how cynicism, as a component of burnout, develops in medical students from their first to their third year in medical school. Firstly, we applied latent growth modeling and saw an increase in cynicism among medical students as the training progresses. Secondly, we were interested in possible different developmental patterns of cynicism, thus applied latent class growth modeling and growth mixture modeling. Our sample was classified into a *high-level* group (34%) and an *increasing* group (66%). The *high-level* group experienced relatively stable heightened cynicism, whereas in the *increasing* group cynicism gradually developed over the three annual measurements. Thirdly, we examined the relationships between obtained classified groups with different trajectory patterns of cynicism and the applicability of signature character strengths, SWB, and PWB (autonomy, engagement, mastery, meaning, optimism, and relationship). Medical students overall reported higher scores on applicability of signature character strengths in private life than during studies over the whole measurement period. Furthermore, students in the *increasing* group experienced significantly higher scores of PWB, mastery, optimism, and relationship at the first measurement compared to the *high-level* group.

Regarding our first hypothesis, concerning the gradual development of cynicism as found in our study’s sample, one explanation could be provided by the training process and learning environment (formal, informal, and hidden curriculum) itself. It may contribute to the deterioration of mental health, quality of life (Klier, 2010; Brazeau et al., 2014) and empathy decline (Neumann et al., 2011) in medical students. According to several studies, there is evidence that these problems, continuously pervading this profession, have their origin in medical education (West et al., 2006; West and Shanafelt, 2007; Dyrbye et al., 2010). Regrettably, during medical school interpersonal skills linked to being an active member of an institution are underrepresented in curricula, compared to the heavily emphasized training of technical skills linked with being a physician (Montgomery et al., 2013). The professional identity of medical students and their perception of what includes “bad and good doctoring” (e.g., professionalism) are shaped during the time in medical school (Hafferty, 1998; Elliott et al., 2009). They experience a learning environment characterized by formal, informal, and hidden aspects, contributing significantly to this identity-shaping process (Hafler et al., 2011). While professionalism may be valued in the formal curriculum, the hidden curriculum values competitive or performance-oriented behavior over collaboration. The hidden curriculum is a huge part of the learning process, comprising e.g., customs, rituals or aspects that are taken for granted. A hidden curriculum is defined as a conglomeration of cultural influences, which are conveyed through structural or institutional levels (Hafferty, 1998). Feudtner et al. (1994) stated, there is an increase in cynicism and development of ethical erosion due to the contribution of the hidden curriculum. In this regard, Arthur et al. (2015) highlighted the importance of implementing character strengths and virtue training in the formal curricula at universities and informal training by senior staff to help resolve ethical dilemmas which physicians are faced with. Thus, the moral character of good doctors can be formed, in order to contribute to the health of patients and doctors.

Concerning the development of cynicism, one could further argue that not all freshmen at the medical university are used to growing pressure, limited support or hierarchical structures. Thus, having limited resources to deal with new and challenging circumstances may result in a growing cynical attitude toward your own studies, resulting in emotional exhaustion or spill over to other domains of life, negatively affecting them (Bergmann et al., 2019). Therefore, students are encouraged to find healthy coping strategies (e.g., personally reflection, peer support) or strong mentors to protect themselves against this negative trend (Cohen et al., 2009). Moreover, not only students are asked to maintain their own well-being, also universities should be held responsible. Since guidelines for medical schools are limited, Kemp et al. (2019) have taken up this issue and provided comprehensive recommendations for an educational program in order to promote well-being and learning in medical students. They do not only recommend addressing changes in the curricula, but further expanding on students’ selection process, learning environment and staff selection. Although they provide a wide range of supporting documents, a sound evidence base developed by longitudinal data is still needed.

According to our analysis, there are two different medical student groups regarding cynicism development. How can it be explained that one part of the students is more cynical at the beginning of their studies (*high-level* group) and others become cynical over time (*increasing* group)? As Moir et al. (2018) emphasized, traits of medical students can play an important role in deteriorating mental health. Therefore, it might be conceivable that students from the *high-level* group possess more of a cynicism trait-like characteristic, compared to students in the *increasing* group. Examining our two groups, this would mean that some students are quite stable in their cynicism values (as seen in the *high-level* group), whereas other students’ cynicism scores are more adaptable over time and situations. At the beginning of the second year, medical students start to gather first experiences with patients which may be leading to a divergence between expected and perceived experiences in the health care setting. The ones having preliminary lower cynical attitudes toward their studies might also have higher expectations regarding its potential usefulness in the beginning. Therefore, some students might perhaps become disillusioned as they progress through medical training. In this regard, it might be beneficial while being in the phase of occupational orientation (e.g., in schools, counseling facilities, during open days, etc.) to present contents and difficulties to be expected during medical studies, so that interested young adults get a more realistic picture of what they can expect during medical school and from first patient interactions.

Despite being a rather robust statistical method, whenever assumptions of the mixed ANOVAs were not met, they were subsequently corrected. Facing violations of sphericity assumptions and heteroscedasticity between the *high-level* and the *increasing* group were present in several variables (e.g., applicability of signature character strengths in private life). The violation of these requirements can stem from unequal group sizes or within group variation of the respective variable. If not corrected, this could lead to false positive result interpretation (Type I error). Although, corrected for violated assumptions of mixed ANOVA, it cannot be ruled out that the data may contain other information that was not discovered by our analyses.

Our findings revealed that both groups generally rated the applicability of signature character strengths in private life higher than during studies. Huber et al. (2019) reported similar results for a sample of physicians. These findings could be explained by the fact that character strengths and their application are not mentioned or trained in the medical curriculum *per se*. Consequently, medical students often do not know their character strengths and how they can be applied in the context of study whereas in private context they might apply them intuitively. One further explanation for this could be the standardized curriculum, which mainly consists of lectures in the preclinical years. Therefore, students may have had fewer opportunities to apply their character strengths in the specific academic context. In particular, the applicability of signature character strengths in private life in the *high-level* group increased from initial to the last measurement, which might be explained by the possibility that these students seek more activities in their private lives that build upon their character strengths. In addition, it is conceivable that the benefits of applying one’s signature character strengths in the private context can spill over to the study-related context, thus potentially affecting the cynical attitude, as can be seen by significantly decreasing cynicism between T1 and T2 in the *high-level* group. Since character strengths are related to stress coping behavior (Harzer and Ruch, 2015) and health (Proyer et al., 2014), we argue that the applicability of signature character strengths may play a role in influencing the development of cynicism, as it reflects the opportunity to deploy personal resources, thus being beneficial for the academic performance of medical students (Trucchia et al., 2013) and potentially buffering harsh and adverse influences occurring during medical training.

Apart from the applicability of signature character strengths, components of well-being may also play a vital role in the developmental process of cynicism. Our results showed optimism to be significantly higher at the initial measurement in the *increasing* group compared to the *high-level* group. Optimism may play a role in developing cynicism at the time of entering medical school, which would be in line with Chang et al. (2000), who found optimism to be negatively related to cynicism. Concerning the PWB components mastery and relationship, one could argue that students with higher values are less cynical. This would be comparable to results by Bentea (2017), who found teachers with lower levels of mastery and relationship having higher levels of cynicism. Overall, well-being proved to be significantly different between both groups at initial measurement. Training well-being of medical students is important as it is directly related to better academic performance (Trucchia et al., 2013), reduced stress levels (Rosenzweig et al., 2003), empathy (Thomas et al., 2007), resilience and patient care (Dunn et al., 2008). Our results underscore the importance of early interventions that may contribute to students’ well-being to prevent a continuing decline or failing increase.

### Limitations and Future Research

Finally, a number of important limitations need to be considered:

Since our longitudinal study was explorative, it cannot provide causal relations between observed variables. Growth mixture modeling has become of increasing interest to researchers in psychology in recent years. GMM is in several regards a useful exploratory procedure (e.g., for identifying unobserved subpopulations), but it has its constraints. In general, mixture models account for non-normality in observed data. Non-normality may occur due to poor measurement, mixed groups of individuals or true non-normally distributed data. Therefore, finding false unobserved groups by misinterpreting GMM results is a real danger (Ram and Grimm, 2009). Although usually large samples are recommended for SEM and GMM, there is no general rule regarding the necessary minimum sample size (Ram and Grimm, 2009). The required sample size depends on many factors (e.g., number of latent classes, model size, distribution and reliability of the variables), but using small sample sizes in growth models may result in the reduction of power (Muthén and Muthén, 2002). Furthermore, the relatively small sample size can lead to attrition biased data interpretation, which is a common problem in longitudinal research (e.g., Goodman, 1996). This selection process can often result in reduced variance (Pearson, 1903) and therefore, may lead to a biased (e.g., homogenous) sample or might limit the generalizability of this data set. For example, participants who continued with all three annual measurements may have other characteristics (e.g., persistence) compared to people who dropped out earlier (e.g., questionnaire was too long for them, reward not satisfying enough, etc.). Moreover, the participants in our sample were chosen from one medical university only. These problems (e.g., misinterpretation, and loss of power) can be addressed by replicating results with new data. Thus, future studies should investigate larger samples of medical students at other universities in order to validate the results obtained in our study. Future research might also take universities from different countries and cultural backgrounds into account with large, multi-centered studies including students from other subjects in order to obtain generalizable results. Since we did not measure the influence of the formal, informal or hidden curriculum on the development of cynicism, studies are necessary that allow their examination. Moreover, studies should identify other personal and training-related features influencing cynicism.

We further measured the applicability of character strengths, based on each individual’s top five character strengths. The top five character strengths differed from student to student and are spread amongst the six virtues. It is conceivable that this approach has influenced our results. In a similar medical sample, Hausler et al. (2017b) found stronger relations between happiness-related strengths (hope, zest, gratitude, curiosity, and love) and components of well-being than with other character strengths. Thus, medical students in our sample who shared this happiness-related strengths profile may generally experience higher levels of well-being and lower levels of cynicism. It might be possible that participants with signature character strengths originating from different virtues be distinct from those who have a strengths profile from one single virtue. Further, it would be interesting to see if students fitting the character strengths profile required in the context of medical studies have different health-related outcomes compared with students who do not match this profile. Therefore, future studies should take up this idea to facilitate a better understanding of the underlying mechanisms. Merritt et al. (2018) found that the daily variation of the application of signature character strengths is linked to daily strain and daily job satisfaction, whereas global strength use was associated with global strain and global job satisfaction. This may also relate to medical students. In this context, it would be useful to assess intervention programs regarding their effectivity to help students to identify and apply their signature character strengths. Furthermore, implementing well-being curricula for medical students may be reasonable, since improved well-being of physicians has positive effects on reflective practice, empathy and communication skills (Neumann et al., 2011). Focusing on strengths interventions, Niemiec (2014) provides an extensive strategy in implementing mindfulness-based strength practice in the training of physicians.

## Conclusion

In conclusion, our study has the following implications:

This explorative study has shown that medical students in Austria experience an increase of cynicism during their 3-year preclinical education in medical school. Two different patterns of cynicism development were identified: *high-level* vs. *increasing*. Data showed that students from both groups perceived lower applicability of signature character strengths, in particular during studies. Moreover, students allocated to the group with continuously higher levels of cynicism reported lower PWB (mastery, optimism, and relationship). Broad strategies for identifying character strengths and implementing well-being as well as character trainings into curricula and workplace culture may be a beneficial approach, in order to avoid early onset of burnout-related symptoms. These strategies should consider characteristics of the targeted individuals/workplaces and need to be empirically tested. This research project expands on the knowledge of the developmental process of cynicism as a burnout burnout-related symptom in medical students and its relations to the applicability of signature character strengths and well-being.

## Data Availability Statement

The datasets generated for this study are available on request to the corresponding author.

## Ethics Statement

This study was carried out in accordance with the recommendations of the Board for Ethical Questions in Science of the University of Innsbruck with written informed consent from all subjects. All subjects gave written informed consent in accordance with the Declaration of Helsinki. The protocol was approved by the Board for Ethical Questions in Science of the University of Innsbruck.

## Author Contributions

AH, CS, TH, and SH were substantially involved in the planning and conducting of the study. TK carried out the data analysis and the drafting of the manuscript. All authors revised the manuscript critically for important intellectual content, and read and approved the accepted version.

## Conflict of Interest

The authors declare that the research was conducted in the absence of any commercial or financial relationships that could be construed as a potential conflict of interest.
